# Assessment of Functionality, Global Clinical Impression, and Addiction Severity in Patients with Dual Diagnosis Treated with Long-Acting Injectable Antipsychotics in a Specialized Center in Bogotá-Colombia

**DOI:** 10.1192/j.eurpsy.2025.1202

**Published:** 2025-08-26

**Authors:** J. Gonzalez Giraldo, A. Cruz

**Affiliations:** 1 Campo Victoria, Grupo Cisne, Bogota, Colombia

## Abstract

**Introduction:**

Dual diagnosis, involving both a mental disorder and an addiction disorder, significantly impacts patients’ functionality and quality of life. In Colombia, from 2009 to 2017, an average of 20,590 individuals with dual diagnosis were treated annually, representing 7.2 Disability-Adjusted Life Years (DALYs) (Ministerio de Salud, Boletín de Salud Mental No. 7, 2018). This study investigates effective therapeutic strategies for this complex condition by examining six patients with schizophrenia or other psychotic disorders and comorbid addiction who were treated with depot antipsychotics. We assessed their personal and social functioning, symptom severity using various surveys.

**Objectives:**

Describe outcomes of long-acting injectable antipsychotics in patients with dual diagnosis beyond the clinic

To promote research involving a larger number of subjects with dual diagnosis and co-occurring mental disorders, like schizophrenia

Continue the pursuit of tools aimed at improving the quality of life in patients with dual diagnosis.

**Methods:**

This case series involved six patients, all men, aged 20-40 years, all of them using depot antipsychotics (paliperidone quarterly = 2, paliperidone monthly = 3, and risperidone biweekly = 1), followed over 12 months in an addiction program with quarterly evaluations (T0=initial contact, T1=three months, T2=six months, T3=nine months, T4=twelve months) from July 2022 to August 2024 on an outpatient basis. All participants had an addictive disorder and schizophrenic and schizoaffective disorder diagnosed. We administered four periodic surveys (ASSIST, CGI-I, PSP) to evaluate personal and social functioning and symptom severity. Results were collected in a database through in-person visits or telephone contact, with surveys conducted by a multidisciplinary team including psychology, social work, occupational therapy, and psychiatry.

**Results:**

the ASSIST scores improved from [T0=1.333 (44.43%) to T4=2 (44.67%)], CGI-I scores decreased from [T0=4.33 (61.9%) to T4=3 (42.8%)], and the PSP scale showed improvement from [T0=1.666 (55.53%) to T4=2.166 (72.20%)]. These results reflect enhanced cognitive and social functions, particularly in social functioning. Similar results were observed in patients with schizophrenia in a study by Shi et al. (Neuropsychiatr Dis Treat. 2016;12:2095-2104), supporting the potential benefits of depot antipsychotics for dual diagnosis patients.

**Conclusions:**

Depot antipsychotics can be a valuable tool for managing dual diagnosis, enhancing treatment adherence, general functionality and reducing symptom severity. However, this observational study did not account for substance types or socioeconomic factors.

**Disclosure of Interest:**

None Declared

